# Pathogenic Analysis of Two *SLC22A5* Variants That Alter RNA Splicing in Patients with Primary Carnitine Deficiency

**DOI:** 10.3390/ijns12010017

**Published:** 2026-03-16

**Authors:** Yiming Lin, Yanru Chen, Weihua Lin, Faming Zheng

**Affiliations:** 1 Department of Clinical Laboratory, Quanzhou Maternity and Children’s Hospital, School of Medicine, Huaqiao University, Quanzhou 362000, China; 22192325@163.com; 2 Center of Neonatal Disease Screening, Quanzhou Maternity and Children’s Hospital, School of Medicine, Huaqiao University, Quanzhou 362000, China; wrightlym@sina.com (Y.C.); linweihua2010@sina.com (W.L.)

**Keywords:** primary carnitine deficiency, *SLC22A5* gene, synonymous variant, RNA splicing, minigene

## Abstract

Functional analysis of *SLC22A5* variants can improve diagnostic accuracy in patients with primary carnitine deficiency (PCD). Herein, we performed a genetic analysis of three neonates with PCD. Two of the patients harbored a novel synonymous *SLC22A5* variant that has not been previously reported, and the other patient harbored a classical splice site variant. The splicing patterns of the two *SLC22A5* variants were evaluated using three in silico tools, and in vitro minigene analysis was performed to verify the impact of variants on RNA splicing mechanisms. All three in silico tools predicted that both *SLC22A5* variants could alter normal RNA splicing. Functional studies using minigene assays demonstrated that the c.450C>T (p.F150=) leads to partial exon 2 skipping, and c.394-1G>A leads to intron 1 retention and exon 2 skipping. Intron 1 retention of 65 nucleotides and exon 2 skipping were confirmed by sequencing cDNA amplification products. These results, along with functional evidence, led to reclassification of c.450C>T (p.F150=) and c.394-1G>A as likely pathogenic and pathogenic, respectively. This is the first reported synonymous variant in the *SLC22A5* gene that has been functionally validated to affect RNA splicing, thus enriching the variant spectrum of *SLC22A5* and aiding accurate PCD diagnosis.

## 1. Introduction

Primary carnitine deficiency (PCD, OMIM #212140) is an autosomal recessive metabolic disorder caused by pathogenic variants in the *SLC22A5* gene, which encodes the organic cation transporter novel family member 2 (OCTN2) [1]. OCTN2 plays a critical role in maintaining intracellular carnitine levels by mediating carnitine uptake into cells, including the renal tubular reabsorption of carnitine. Functional defects in OCTN2 typically lead to insufficient cellular carnitine uptake and low cytoplasmic concentrations [2,3,4]. PCD presents with a wide spectrum of clinical phenotypes. Patients diagnosed based on clinical symptoms may exhibit muscle weakness, cardiomyopathy, and arrhythmia, whereas most individuals identified through newborn screening (NBS) remain asymptomatic at the time of diagnosis [5,6,7]. The confirmatory diagnosis of PCD is made through genetic testing and/or carnitine transporter activity analysis. Owing to the complexity of determining carnitine transporter activity, sequencing of *SLC22A5* is currently the first-line detection method for the diagnosis of PCD. Patients can achieve optimal clinical outcomes through early diagnosis and timely supplementation with L-carnitine [8].

*SLC22A5* (MIM# 603377) is located on chromosome 5q31, spans approximately 30 kb, and contains 10 exons. Over 200 pathogenic variants have been reported, with c.51C>G (p.F17L), c.760C>T (p.R254*), and c.1400C>G (p.S467C) being the three most common variants in China [9,10,11]. Numerous variants of *SLC22A5* have been identified using high-throughput sequencing. Interpreting the clinical significance of *SLC22A5* variants is challenging. Although in silico tools are available to predict the functional impact of encoded proteins, these predictions are not always reliable, with many novel variants classified as variants of unknown significance (VUS). Some pathogenic synonymous variants are not usually regarded as deleterious and are frequently missed during genetic testing because of lack of functional data [12,13,14]. Therefore, determining the effects of *SLC22A5* variants on OCTN2 function is of particular importance.

Herein, we present three patients with PCD with low C0 levels identified through NBS, all of whom had compound heterozygous variants of *SLC22A5*. Two of the patients harbored a novel synonymous variant that has not been previously reported, and the other harbored a classical splice site variant. To determine the effect of the two *SLC22A5* variants on RNA splicing mechanisms, in vitro minigene analysis was performed. The results demonstrated that c.450C>T (p.F150=) causes partial skipping of exon 2, whereas c.394-1G>A results in intron 1 retention and exon 2 skipping, thereby explaining the persistently low C0 levels in the affected patients.

## 2. Materials and Methods

### 2.1. Study Cohort

Three patients with PCD identified by NBS were enrolled; one of these patients (no. 3) was described in our previous study [15]. Neonatal dried blood spot specimens were collected and analyzed using AQCUTY TQD and TQS Micro tandem mass spectrometers (Waters, Milford, MA, USA) at the Newborn Screening Laboratory of Quanzhou Maternity and Children’s Hospital. Targeted next-generation sequencing was performed to identify pathogenic variants and confirmed by Sanger sequencing as previously described [16]. Written informed consent was obtained from all patients’ parents. This study was approved by the Ethics Committee of the Quanzhou Maternity and Children’s Hospital (No. 2025-IRB-07, approved on 13 February 2025).

### 2.2. In Silico Analysis

Three in silico tools (SpliceAI, https://spliceailookup.broadinstitute.org/ (accessed on 13 August 2025); RDDC, https://rddc.tsinghua-gd.org/tool/rna-splicer (accessed on 13 August 2025); and ESE Finder 3.0, https://esefinder.ahc.umn.edu/cgi-bin/tools/ESE3/esefinder.cgi (accessed on 13 August 2025)) were used to predict the effect of variants on RNA splicing.

### 2.3. In Vitro Minigene Assays

In vitro minigene assays were conducted using the pcMINI-C vector backbone to analyze the splicing patterns of the wild-type (WT) and *SLC22A5* mutant variants (c.450C>T and c.394-1G>A). Primers containing *BamHI* and *XhoI* restriction enzyme sites were designed to amplify the target fragments, including *SLC22A5* exons 2 and 3 and their flanking sequences (reference transcript NM_003060). The minigene pcMINI-C-SLC22A5-WT/MUT was constructed by inserting intron 1 (583 bp), exon 2 (104 bp), part of intron 2 (864 bp), and exon 3 (155 bp) fragments into the pcMINI-C plasmid vector, which contained the universal exon A-intron A-MCS (multiple cloning sites). Both WT and mutant minigene plasmids were transformed into *Escherichia coli* DH5α-competent cells (DL1080, WEIDI, Shanghai, China); the constructed WT and mutant sequences were confirmed by Sanger sequencing. The constructs were then transfected into human embryonic kidney 293 cells and HeLa cells using Hieff Trans^®^ Liposomal 2000 Transfection Reagent (40802ES03, YEASEN, Shanghai, China) following the manufacturer’s protocol. After 48 h of transfection, the cells were harvested, and total RNA was extracted and reverse transcribed into complementary DNAs (cDNAs) using TransScript Reverse Transcriptase (RR092B, Takara, Kusatsu, Japan). The cDNAs were amplified using polymerase chain reaction with paired primers, and the amplification products were visualized on a 1.5% agarose gel. DNA was extracted from the bands and sequenced using an ABI3730xl sequencer (Applied Biosystems, Foster City, CA, USA). All normal and aberrant splice transcripts were analyzed. All primer sequences are listed in the Supplementary Tables S1 and S2.

### 2.4. Statistical Analyses

Grayscale value analysis was conducted for c.450C>T (p.F150=) using image J 1.53c. The obtained grayscale data were processed using GraphPad Prism 8 and converted into bar charts. All experiments were independently repeated three times. *t*-tests and nonparametric tests were used for statistical comparisons. Differences between groups were considered significant at *p* < 0.05.

## 3. Results

### 3.1. Data of Patients with PCD

All three patients were found to carry biallelic pathogenic variants in the *SLC22A5* gene, which resulted in low C0 levels detected during NBS and at follow-up recall evaluation. All three patients received L-carnitine treatment (100–400 mg/kg/day) and were routinely followed up; no clinical symptoms were observed. The C0 concentrations in patients no. 1 and no. 2 increased significantly after initial treatment (Table 1). After the patients voluntarily stopped L-carnitine supplementation during the follow-up period, the C0 levels of patient no. 2 decreased to 1.89 and 2.97 μmol/L (cutoff value: 9–50 μmol/L) at the ages of 7 months and 3 years and 6 months, respectively. Similarly, the C0 level of patient no. 3 dropped to 2.91 μmol/L (cutoff value: 9–50 μmol/L) at the age of 2 years and 5 months. In each case, L-carnitine supplementation had been stopped 1 year prior to the extended follow-up.

### 3.2. Prediction of Variant Pathogenicity

SpliceAI predicted that c.450C>T (p.F150=) reduces the confidence score of the original 5′ splice donor site by 0.39 and that of the original 3′ splice acceptor site by 0.40, suggesting altered RNA splicing. RDDC predicted that c.450C>T (p.F150=) would produce a novel 5′ splice donor site, which may result in exon 2 skipping. ESE Finder 3.0 further predicted that c.450C>T (p.F150=) would disrupt the binding of exonic splicing enhancer (ESE) to SC35 and SRp40 proteins. Furthermore, SpliceAI predicted that c.394-1G>A reduces the confidence score of the original 5′ splice donor site by 0.86 and that of the original 3′ splice acceptor site by 0.93, suggesting altered RNA splicing. RDDC predicted that c.394-1G>A would produce a novel 5′ splice donor site, which may result in exon 2 skipping. ESE Finder 3.0 further predicted that c.394-1G>A would disrupt the binding of ESE to SF2/ASF and SF2/ASF (IgM-BRCA1) proteins. The prediction results are shown in the Supplementary Table S3.

### 3.3. Functional Study by Minigene Assays

**Figure 1:**
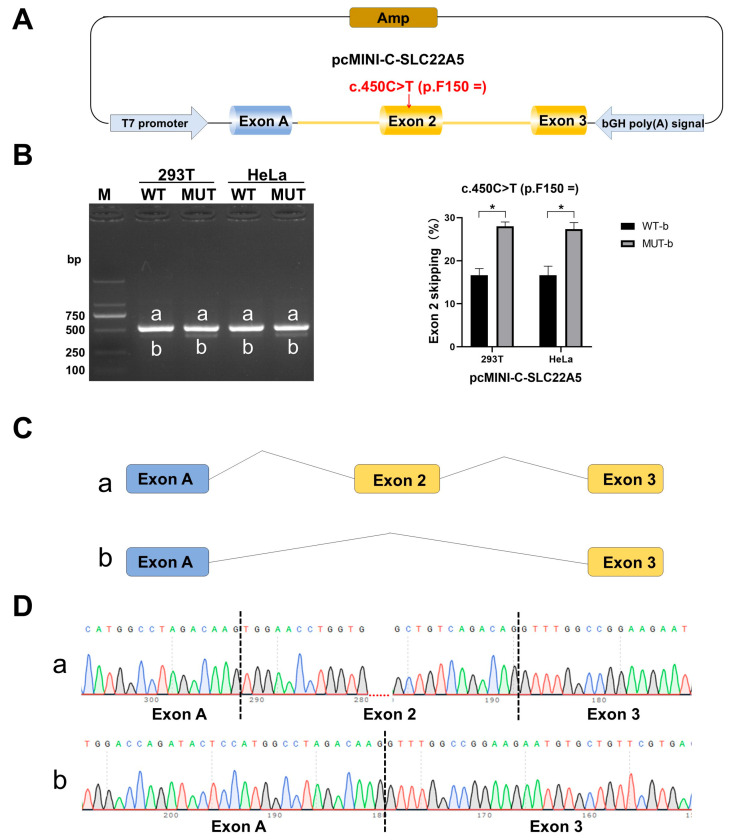
Results of the minigene assay for *SLC22A5* c.450C>T (p.F150=). (**A**) Schematic diagram of cloned *SLC22A5* exons 2 and 3 and the flanking sequences of wild-type (WT) and mutant (MUT) into a reporter vector, pcMINI-C. (**B**) Agarose gel electrophoresis results of reverse transcription polymerase chain reaction products from the pcMINI-C-SLC22A5-WT/MUT constructs. Human embryonic kidney 293 cells (HEK293T) and HeLa cells were used as target cells for transfection. Both WT and MUT vectors have two bands (a and b); in contrast, the proportion of MUT bands (with exon 2 skipping) in the mutant vectors was markedly increased compared with those in WT (n = 3, *p* = 0.0193). Significant differences are indicated by asterisks (* *p* < 0.05) above a bracket connecting two groups. (**C**) Schematic diagram of exon 2 skipping. (**D**) Sanger sequencing of cDNA containing the WT and MUT sequences; the sequence containing exon 2 skipping in MUT transcripts (b) was confirmed.

Agarose gel electrophoresis showed that both WT and mutant vectors had two bands (a and b) with splicing patterns of exon A (192 bp)—exon 2 (104 bp)—exon 3 (155 bp) and exon A (192 bp)—exon 3 (155 bp), respectively. The proportion of shorter bands (exon 2 skipping) in the mutant vectors was markedly higher than that in the WT vector (*p* = 0.0193). These results indicate that c.450C>T (p.F150=) could cause partial exon 2 skipping, leading to an increase in the abundance of aberrant transcripts, thereby affecting normal RNA splicing. Exon 2 skipping was confirmed by sequencing the cDNA amplification products (Figure 1).

**Figure 2:**
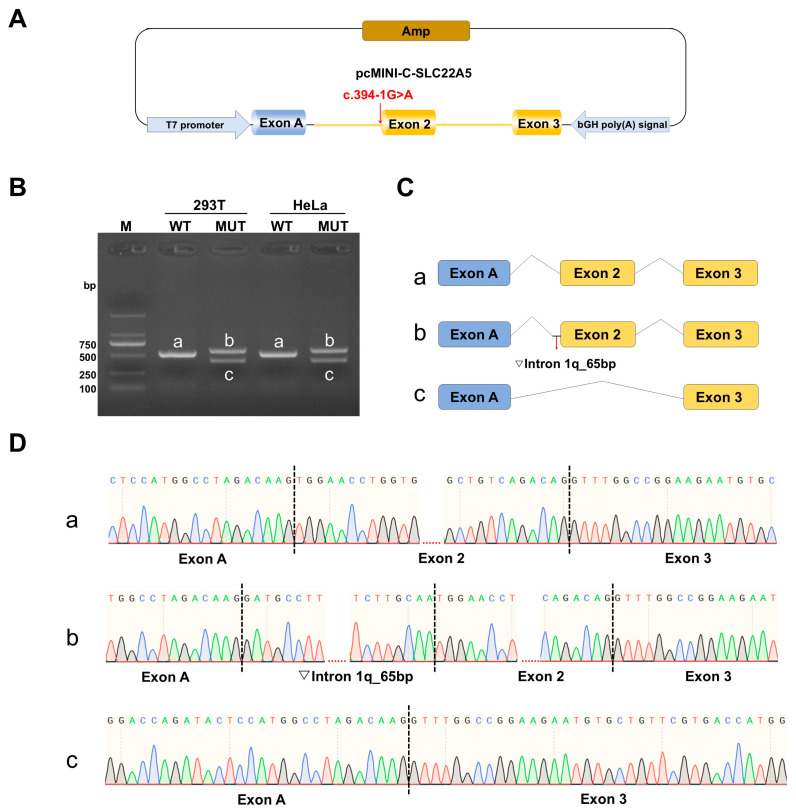
Results of the minigene assay for *SLC22A5* c.394-1G>A. (**A**) Schematic diagram of cloned *SLC22A5* exons 2 and 3 and the flanking sequences of wild-type (WT) and mutant (MUT) into a reporter vector, pcMINI-C. (**B**) Agarose gel electrophoresis results of reverse transcription polymerase chain reaction products from pcMINI-C-SLC22A5-WT/MUT constructs. Human embryonic kidney 293 cells (HEK293T) and HeLa cells were used as target cells for transfection. (**C**) A schematic diagram of intron 1 retention and exon 2 skipping. (**D**) Sanger sequencing of cDNA containing WT (a) and MUT sequences confirmed the retention of 65 nucleotides (GATGCCTTTGCTTTAAAACCTTTTAAAAAGAAGTGAATGATACACCCCCTTTGCTCATCTTGCAA) on the left side of intron 1 (b) and exon 2 skipping (c).

Agarose gel electrophoresis showed that WT vectors produced the expected 521 bp bands (a), with a normal splicing pattern of exon A (192 bp), exon 2 (104 bp), and exon 3 (155 bp). The mutant vectors produced two bands of different sizes (b and c), with splicing patterns of exon A (192 bp)—▽intron 1 (65 bp)—exon 2 (104 bp)—exon 3 (155 bp) and exon A (192 bp)—exon 3 (155 bp), respectively. These results indicate that c.394-1G>A could affect normal RNA splicing, leading to intron 1 retention and exon 2 skipping. The occurrence of exon 2 skipping and the retention of 65 nucleotides (GATGCCTTTGCTTTAAAACCTTTTAAAAAGAAGTGAATGATACACCCCCTTTGCTCATCTTGCAA) on the left side of intron 1 was confirmed by sequencing of the cDNA amplification products (Figure 2).

## 4. Discussion

With the successful and widespread implementation of NBS, an increasing number of patients with PCD and variants in the *SLC22A5* gene have been reported [17]. Our previous study demonstrated that the incidence of PCD in our population was approximately 1 in 10,000 newborns [15]. Recently, a large-scale neonatal genetic screening study involving 29,601 newborns in China showed that the incidence rate of PCD was as high as 1 in 5920 [18]. However, the pathogenicity of many *SLC22A5* variants identified by NBS remains unclear, which not only poses challenges to the diagnosis of PCD but also induces significant anxiety in family members. In this study, two *SLC22A5* variants were confirmed to alter normal RNA splicing. We demonstrated that c.450C>T (p.F150=) leads to partial exon 2 skipping, and c.394-1G>A leads to intron 1 retention and exon 2 skipping, which explains the persistently low C0 levels in the three patients. Thus, our data provide additional evidence supporting the pathogenicity of these two *SLC22A5* variants, contributing to the correct classification of *SLC22A5* variants and accurate diagnosis of PCD.

As this cohort of patients was discovered by NBS and received timely treatment, no clinical symptoms occurred during the follow-up period. However, the biochemical data indicated that patient no. 2 had extremely low C0 concentrations during NBS and follow-up, suggesting that the c.450C>T (p.F150=) variant could lead to impaired OCTN2 function, biochemically confirming the pathogenicity of the variant. Maternal carnitine deficiency could theoretically contribute to transiently low C0 levels in newborns, and we did not perform further measurements of C0 levels in the patients’ parents. However, the persistently low C0 levels observed in patient no. 2 during follow-up ruled out a maternal contribution. Notably, although patients no. 1 and 2 both harbored c.450C>T (p.F150=) with another missense variant, the C0 concentration in patient no. 2 was found to be much lower than that in patient no. 1. We speculate that this is because c.1400C>G (p.S467C) retains relatively high carnitine transport activity. A previous functional study showed that c.1400C>G (p.S467C) had 16.56% normal carnitine transport activity [19]. Although there are no published functional studies of c.338G>A (p.C113Y) in the literature yet, this variant has been identified in several symptomatic patients with PCD, indicating that it could dramatically impair OCTN2 protein function [20,21].

Although amino acid sequences do not change in synonymous variants, the splicing mode of precursor mRNA can be modified; the mis-splicing mediated by synonymous variants is an important pathogenic mechanism for recessive inherited disorders [22,23]. However, these variants are usually classified as likely benign or VUS in the absence of functional studies. No synonymous variants of *SLC22A5* have been reported to date. In the present study, a synonymous variant, c.450C>T (p.F150=), was identified in two patients with PCD; however, the pathogenicity of this variant has not yet been confirmed. This variant is located at the 48th base of exon 2 and is classified as a VUS according to the ACMG guidelines [24]. Bioinformatic analysis using three in silico tools (SpliceAI, RDDC, and ESE Finder 3.0) showed that this variant may alter normal RNA splicing. Given the insufficient accuracy of the prediction tools, in vitro minigene analysis was performed to reveal the impact of c.450C>T (p.F150=) on RNA splicing. Agarose gel electrophoresis showed that the proportion of abnormal splicing bands (exon 2 skipping) in the mutant vectors was markedly increased. Therefore, we can conclude that c.450C>T (p.F150=) leads to partial exon 2 skipping, thereby clarifying the molecular pathogenic mechanism of PCD. To our knowledge, this is the first synonymous variant of the *SLC22A5* gene that has been functionally verified to alter RNA splicing. According to the ACMG guidelines [24], we reclassified this synonymous variant as likely pathogenic based on the following evidence: (i) in vitro minigene analysis verified this variant can lead to abnormal RNA splicing (PS3); (ii) the variant has an extremely low allelic frequency of 0.000005576 in GnomAD (PM2); (iii) both of the two patients with PCD harbored the variant in compound heterozygosity with another known pathogenic variant, and the allelic variants were inherited from their respective parents (PM3); and (iv) the variant was predicted to alter normal RNA splicing by three in silico tools (PP3).

Variations at the canonical acceptor and donor sites usually lead to loss of protein function and are considered pathogenic. c.394-1G>A is located at the first base of intron 1 of *SLC22A5* and has an extremely low allelic frequency (0.000001239) in GnomAD. We noticed that another variant, c.394-1G>T, at the same splice site, was reported in two other Chinese patients [25,26]. Bioinformatics analysis also showed that this variant may alter normal RNA splicing. In vitro minigene analysis was conducted to obtain direct evidence, which confirmed that c.394-1G>A would lead to intron 1 retention and exon 2 skipping. Intron 1 retention of 65 nucleotides was predicted to cause a frameshift, generating an early stop codon PTC within the retained sequence, which may result in a truncated protein of length 142 aa. Exon 2 skipping was predicted to cause a frameshift (c.394_497del p.Trp132ValfsTer28), resulting in a premature stop codon (PTC) within exon 3, which may produce a truncated protein of length 158 aa. On the basis of these results as well as evidence from a functional study, the variant was classified as pathogenic.

In this study, direct analysis of RNA splicing patterns from patient blood samples (in vivo experiments) was not performed to verify the pathogenicity of the two variants; therefore, the actual RNA splicing status in these patients remains unclear. The minigene assay used here, although an in vitro system, is a well-established and widely accepted method for evaluating splicing abnormalities when patient RNA is unobtainable. It has proven to be an effective tool for studying the pathogenic mechanisms of variants. Overall, the experimental results in this study sufficiently confirm the pathogenicity of these two variants.

In addition to molecular splicing analysis, complementary biochemical markers can provide further evidence for PCD diagnosis. Crefcoeur et al. [27] demonstrated that the ratio of urinary C0 to plasma C0 (Ratio U:P) effectively discriminates between true-positive and false-positive referrals from NBS, particularly in unsupplemented infants under 1 month of age. This ratio, which reflects renal carnitine wasting, serves as an effective indicator of OCTN2 dysfunction. Fractional carnitine clearance provides similar information and has been proposed as an early discriminatory test for PCD. Although urine samples were not available for such analysis in this study, we recommend same-day paired plasma and urine collection for calculation of carnitine clearance or the U:P ratio during initial PCD evaluation. This rapid, non-invasive test can support clinical decision-making prior to the availability of genetic and functional study results. Furthermore, measurement of carnitine transporter activity in cultured fibroblasts remains the gold standard for confirming OCTN2 dysfunction, but it requires skin biopsy and is available only in a few specialized laboratories [19,28]. In this study, functional assays of carnitine transport activity in fibroblasts were not performed; thus, the precise transport activity of these two variants remains unknown. However, the combination of clinical, genetic, and minigene functional data strongly supports the deleterious effect of these variants.

## 5. Conclusions

The two *SLC22A5* variants were functionally confirmed to alter normal RNA splicing. We demonstrated that c.450C>T (p.F150=) leads to partial exon 2 skipping, and c.394-1G>A leads to intron 1 retention and exon 2 skipping. To our knowledge, this is the first reported synonymous variant in the *SLC22A5* gene that has been functionally verified to alter RNA splicing, thus enriching the variant spectrum of *SLC22A5*. Our results contribute to a better understanding of the pathogenicity of *SLC22A5* variants, thereby facilitating the precise diagnosis of PCD.

## Figures and Tables

**Table 1:** Data of three patients with primary carnitine deficiency (PCD).

Patients No.	Gender	C0 Levels at NBS (μmol/L) *	C0 Levels at Recall (μmol/L) *	C0 Levels After Initial Treatment (μmol/L) ^#^	Genotype		Clinical Manifestations	Age of Last Follow-Up	References
1	Male	8.94	7.17	57.96	c.1400C>G (p.S467C)	c.450C>T (p.F150=)	None	4 months	This study
2	Female	4.13	3.48	19.85	c.338G>A (p.C113Y)	c.450C>T (p.F150=)	None	6 years and 5 months	This study
3	Female	5.34	6.02	Not available	c.797C>T (p.P266L)	c.394-1G>A	None	7 years and 9 months	Lin et al. [15]

C0: free carnitine, NBS: newborn screening. * The cutoff value of C0 is 9–50 μmol/L. ^#^ The C0 concentrations of patients no. 1 and no. 2 were quantified at 4 months and 1 month old, respectively. Data for patient no. 3 were not available because she did not supplement L-carnitine as required after recall.

## Data Availability

Data are included in the article/Supplementary Materials/references in this article. The datasets used and analyzed in this study are available from the corresponding author upon reasonable request.

## Supplementary Materials

The following supporting information can be downloaded at https://www.mdpi.com/article/10.3390/ijns12010017/s1, Table S1: Primer sequences of c.450C>T (p.F150=) used for minigene assays; Table S2: Primer sequences of c.394-1G>A used for minigene assays; Table S3: Prediction of variant pathogenicity.

## Abbreviations

The following abbreviations are used in this manuscript:

PCD	primary carnitine deficiency
OCTN2	organic cation transporter novel 2
NBS	newborn screening
C0	free carnitine
VUS	variants of unknown significance
WT	wild-type
ESE	exonic splicing enhancer
