# Supplementary material for: Pathogenic Analysis of Two SLC22A5 Variants That Alter RNA Splicing in Patients with Primary Carnitine Deficiency
Source: Int J Neonatal Screen. 2026 Mar 16;12(1):17. doi: 10.3390/ijns12010017 (PMC13026843; doi:10.3390/ijns12010017)
Supplement: Supplementary file 1 [file IJNS-12-00017-s001.zip › IJNS-4147435-supplementary.pdf]

Supplementary Table S1. Primer sequences of c.450C>T (p.F150=) used for minigene assays.

Primer	Sequences of primers
7115-F	ccatccaggttcacgcttat
7395-F	aggcaagggtctgagttcct
9246-R	gacacaacataatggacccctc
9535-R	ctgggtaacaagagtgaaactcc
13500-F	aaaattggcagttggatcca
14593-R	actaagaccaaggagccaca
pcMINI-SLC22A5-BamHI-F	GCTCGGATCCggaagctggaaactcaagcg
SLC22A5-mut-F	CACTCACAATCTCCTTGTTCTTtGTGGGTGTGCTGT
SLC22A5-mut-R	CCAACAGCACACCCACaAAGAACAAGGAGATTGT
pcMINI-SLC22A5-XhoI-R	tttcCTCGAGccactagtggctggtgagagct
pcMINI-C-SLC22A5-BamHI-F	GCTCGGATCCtgagcctagtatagttggtg
pcMINI-C-SLC22A5-XhoI-R	TAGACTCGAGCCCCAGGACAAATGCTGCCACA
pcMINI-C-Joint-F	gaccagcattctctcggttgctgttcacat
pcMINI-C-Joint-R	atgtgaacagcaaccgagagaatgctggtc
pcMINI-F	ACTTAAGCTTatgagtgggctttggggtggccggtt
pcMINI-R	TAGAAGGCACAGTCGAGG
pcMINI-C-F	ACTTAAGCTTatgagtgggctttggggtggccggtt
pcMINI-C-R	TAGAAGGCACAGTCGAGG

Supplementary Table S2. Primer sequences of c.394-1G>A used for minigene assays.

Primer	Sequences of primers
7115-F	ccatccaggttcacgcttat
7395-F	aggcaagggtctgagttcct
9246-R	gacacaacataatggacccctc
9535-R	ctgggtaacaagagtgaaactcc
13500-F	aaaattggcagttggatcca
14593-R	actaagaccaaggagccaca
pcMINI-SLC22A5-BamHI-F	GCTCGGATCCggaagctggaaactcaagcg
SLC22A5-mut-F	ccccttttgctcatcttgcaaTGGAACCTGGTGTGTGAG
SLC22A5-mut-R	CTCACACACCAGGTTCCAttgcaagatgagcaaagggg
pcMINI-SLC22A5-XhoI-R	tttcCTCGAGccactagtggctggtgagagct
pcMINI-C-SLC22A5-BamHI-F	GCTCGGATCCtgagcctagtatagttggtg
pcMINI-C-SLC22A5-XhoI-R	TAGACTCGAGCCCCAGGACAAATGCTGCCACA
pcMINI-C-Joint-F	gaccagcattctctcggttgctgttcacat
pcMINI-C-Joint-R	atgtgaacagcaaccgagagaatgctggtc
pcMINI-F	ACTTAAGCTTatgagtgggctttggggtggccggtt
pcMINI-R	TAGAAGGCACAGTCGAGG
pcMINI-C-F	ACTTAAGCTTatgagtgggctttggggtggccggtt
pcMINI-C-R	TAGAAGGCACAGTCGAGG

Supplementary Table S3. Prediction of variant pathogenicity.

**Nucleotide change**	**SpliceAI**	**RDDC^{SC}**	**ESE Finder 3.0**
c.394-1G>A	WT Acceptor site Loss:0.93<br>WT Donor site Loss:0.86	Splice Pattern 1: Exon 2 deleting 95bp<br>Splice Pattern 2: Exon 2 Skipping	SF2/ASF protein loss<br>SF2/ASF(IgM-BRCA1) protein loss
c.450C>T (p.F150 =)	WT Acceptor site Loss:0.39<br>WT Donor site Loss:0.40	Splice Pattern 1: Exon 2 deleting 95bp<br>Splice Pattern 2: Exon 2 Skipping	SC35 protein loss<br>SRp40 protein loss
